# Immune checkpoint inhibitors in first-line therapies of metastatic or early triple-negative breast cancer: a systematic review and network meta-analysis

**DOI:** 10.3389/fendo.2023.1137464

**Published:** 2023-05-09

**Authors:** Xueyan Liang, Xiaoyu Chen, Huijuan Li, Yan Li

**Affiliations:** ^1^ Phase 1 Clinical Trial Laboratory, Guangxi Academy of Medical Sciences and the People’s Hospital of Guangxi Zhuang Autonomous Region, Nanning, Guangxi, China; ^2^ Department of Pharmacy, Guangxi Academy of Medical Sciences and the People’s Hospital of Guangxi Zhuang Autonomous Region, Nanning, Guangxi, China

**Keywords:** immune checkpoint inhibitors, atezolizumab, pembrolizumab, durvalumab, triple-negative breast cancer, network meta-analysis, anti-PD1/PD-L1

## Abstract

**Background:**

The optimal first-line immune checkpoint inhibitor (ICI) treatment strategy for metastatic or early triple-negative breast cancer (TNBC) has not yet been determined as a result of various randomized controlled trials (RCTs). The purpose of this study was to compare the efficacy and safety of ICIs in patients with metastatic or early TNBC.

**Methods:**

RCTs comparing the efficacy and safety of ICIs in patients with TNBC were included in the studies. Based on PRISMA guidelines, we estimated pooled hazard ratios (HRs) and odds ratios (ORs) using random-effects models of Bayesian network meta-analysis. Primary outcomes were progression-free survival (PFS) and overall survival (OS). Secondary outcomes included pathologic complete response rate (pCR), grade ≥ 3 treatment-related adverse events (trAEs), immune-related adverse events (irAEs), and grade ≥ 3 irAEs.

**Results:**

The criteria for eligibility were met by a total of eight RCTs involving 4,589 patients with TNBC. When ICIs were used in patients without programmed death-ligand 1 (PD-L1) selection, there was a trend toward improved PFS, OS, and pCR, without significant differences. Pembrolizumab plus chemotherapy is superior to other treatment regimens in terms of survival for TNBC patients based on Bayesian ranking profiles. Subgroup analysis by PD-L1 positive population indicated similar results, and atezolizumab plus chemotherapy provided better survival outcomes. Among grade ≥ 3 trAEs and any grade irAEs, there was no statistically significant difference among different ICI agents. The combination of ICIs with chemotherapy was associated with a higher incidence of grade ≥ 3 irAEs. Based on rank probability, the ICI plus chemotherapy group was more likely to be associated with grade ≥ 3 trAEs, any grade irAEs, and grade ≥ 3 irAEs. Hypothyroidism and hyperthyroidism were the most frequent irAEs in patients receiving ICI.

**Conclusions:**

ICI regimens had relatively greater efficacy and safety profile. Pembrolizumab plus chemotherapy and atezolizumab plus chemotherapy seem to be superior first-line treatments for intention-to-treat and PD-L1-positive TNBC patients, respectively. It may be useful for making clinical decisions to evaluate the efficacy and safety of different ICIs based on our study.

**Systematic review registration:**

https://www.crd.york.ac.uk/PROSPERO/, identifier CRD42022354643.

## Introduction

The triple-negative breast cancer (TNBC) is associated with aggressive histology, a poor prognosis, and nonresponsiveness to hormonal therapy (1). High-risk early breast cancer (BC) with a neoadjuvant treatment is used widely to shrink tumors and reduce their size. Currently, the most common neoadjuvant therapies include chemotherapy, anti-human epidermal growth receptor 2 (HER2) therapy, endocrine therapy, and the co-administration of chemotherapy and HER2. Despite the lack of an anti-HER2 therapy and the potential antagonism between endocrine therapy and chemotherapy, anthracycline plus cyclophosphamide plus taxane neoadjuvant chemotherapy remains the preferred treatment option for patients with TNBC (2, 3). Despite the lack of clear overall survival (OS) benefits, bevacizumab is commonly used in combination with chemotherapy as a maintenance therapy in several countries (4, 5).

There is a poor survival outcome among patients with TNBC following standard neoadjuvant chemotherapy (6). To further increase survival outcomes for patients with TNBC, new strategies and agents are urgently needed. There has been a significant amount of research on the role of immune checkpoint molecules in preventing immune system suppression in tumor microenvironments (7). These molecules include cytotoxic T lymphocyte antigen 4 (CTLA-4), programmed cell death 1 (PD-1), and programmed cell death ligand 1 (PD-L1). The effect of these molecules on tumor cells and/or tumor-infiltrating lymphocytes (TILs) in metastatic TNBC has been evaluated (7). Research on immune checkpoint inhibitors (ICIs) has been a major focus in the last few years for patients with TNBC. Based on the updated guidelines, ICI plus chemotherapy is recommended as a treatment option for TNBC (8, 9). An important characteristic of cancer is its ability to evade immune destruction, which has led to the identification of escape mechanisms for cancer cells. Immunotherapeutic approaches to TNBC are rationalized by several factors. There is a strong correlation between a high level of TILs in TNBC and a positive response to ICI in other types of cancer (10). Additionally, immune evasion molecules such as PD-L1 are highly expressed in both tumors and immune cells (11), and the presence of many non-synonymous mutations that produce tumor-specific neoantigens may enhance the anti-tumor immune response and enhance the rationale for ICI treatment (12, 13). The treatment of early and metastatic TNBC using immunotherapeutic agents such as atezolizumab, durvalumab, and pembrolizumab showed that ICI had a superior effect in earlier treatment lines as well as in tumors positive for PD-L1 (14–21). TNBC poses a challenge for treatment because there are no specific targets for therapeutic intervention (22). Although previous published RCTs showed the superior efficiency of these novel ICIs for TNBC and the efficacy of recommended ICIs for TNBC has mainly been evaluated in clinical trials, there remains a lack of evidence to evaluate the relative effectiveness and safety according to classes of immunotherapies and targeted therapies.

Traditional pairwise meta-analysis can only compare two drug classes that have already been evaluated in previously published studies. However, the optimal types of ICIs for the treatment of TNBC are various, and the efficacy and safety of different types of ICIs remain inconclusive. In a complex choice with several optional strategies for treatment, and some therapeutic strategies have not been directly compared, a Bayesian network meta-analysis (NMA) can compare direct and indirect comparisons of different treatment strategies simultaneously within a single network and rank optional treatments according to comparative efficacy and safety. The 95% intervals generated by the Bayesian NMA are slightly wider than those under the traditional pairwise meta-analysis, and the primary reasons including the size of the discrepancies was heavily dependent on the number of included studies and the heterogeneity of the results, another key driver for the difference was the “prior information” used in the Bayesian analyses for random-effects model. However, from a practical standpoint, most health technology assessment bodies see little harm in being conservative by risking an overestimation of the width of 95% intervals as opposed to risking underestimation. Considering this background, we performed the present systematic review and Bayesian NMA of randomized controlled trials (RCTs) that assessed the efficacy and safety of ICI in patients with TNBC. In this study, we aim to determine the magnitude of benefit and safety that can be derived from different types of first-line ICIs in order to guide decision-making in clinical practice and future research on novel immunotherapeutic agents.

## Methods

In this study, we followed the Preferred Reporting Items for Systematic Reviews and Meta-Analyses (PRISMA) guidelines with an extension for NMAs (23). The study protocol was registered with PROSPERO (CRD42022354643).

### Data sources and search strategy

MEDLINE (via OVID SP), Embase (via OVID SP), and PubMed (via OVID SP) databases were searched systematically for articles published up to October 8, 2022. The following search terms were used: breast cancer, TNBC, immune checkpoint inhibitor, programmed cell death protein-1, PD-1, programmed death ligand 1, PD-L1, atezolizumab, durvalumab, nivolumab, pembrolizumab, cytotoxic T-lymphocyte-associated antigen 4, CTLA-4, ipilimumab, and the name of other ICIs. We identified studies that were eligible and did not impose any language restrictions. We reviewed the reference lists of the identified trials, reviews, and meta-analyses in order to identify additional resources. Trials enrolled in the study were approved by all authors.

### Selection criteria

Potentially eligible studies had to satisfy the following criteria in order to be included in the systematic review: (i) RCTs that study evaluated ICI, (ii) in TNBC patients previously untreated with ICI and (iii) with available results on primary outcomes or secondary outcomes. We looked at the two primary outcomes included OS and progression-free survival (PFS), as well as the secondary outcomes, included pathological complete response (pCR), grade ≥ 3 treatment-related adverse events (trAEs), immune-related adverse events (irAEs), and grade ≥ 3 irAEs. Exclusion criteria were: (i) non-RCTs studies such as single-arm trials or retrospective studies, (ii) no ICI treatments were used in the treatment arm of these trials, and (iii) the literature search was conducted in the context of ongoing studies with unpublished results. The analysis included all studies that met the inclusion criteria.

### Data extraction

The following variables were extracted if available: name of the clinical trial, name of the first author, year of publication, study sample size, ICI used in combination with chemotherapy, chemotherapy regimen used as a control arm, and clinical outcomes of intention-to-treat (ITT) and PD-L1 status subgroups, including median and hazard ratios (HRs) and 95% confidence intervals (CIs) for PFS and OS, and the incidence of pCR and AEs. A message was sent by e-mail to the author when a primary or secondary outcome had not been reported.

### Assessment of the risks of bias

In order to assess the methodological quality of the included RCTs, the Risk of Bias 2 tool was used to grade each trial as having low bias, high bias, or some concerns regarding bias (24).

### Statistical analysis

In this study, we compared multiple trials involving different ICIs for the treatment of TNBC using NMA. Random-effects models were used to pool outcome measures. In the random-effects model approach, it is assumed that different studies estimate effects that are related to intervention but differ from each other. By using this approach, we are able to explain heterogeneity that cannot be simply explained by other factors. OS and PFS outcomes were expressed as HRs with 95% CIs. pCR, grade ≥ 3 trAEs any grade irAEs, and grade ≥ 3 irAEs were expressed as odds ratios (OR) and 95% CIs.

Bayesian NMA was performed using WinBUGS 1.4.3 software (MRC Biostatistics Unit, Cambridge, UK). It was conducted for each treatment comparison 100,000 times, with the first 10,000 iterations being discarded. To estimate the rank probabilities and assess the likelihood of each treatment regimen from best to worst, we used the surface under the cumulative ranking probabilities. As a result of the connections between the included studies and the number of participants, network plots were constructed based on the number of studies and participants.

## Results

### Systematic review and characteristics of selected trials

**Figure 1:**
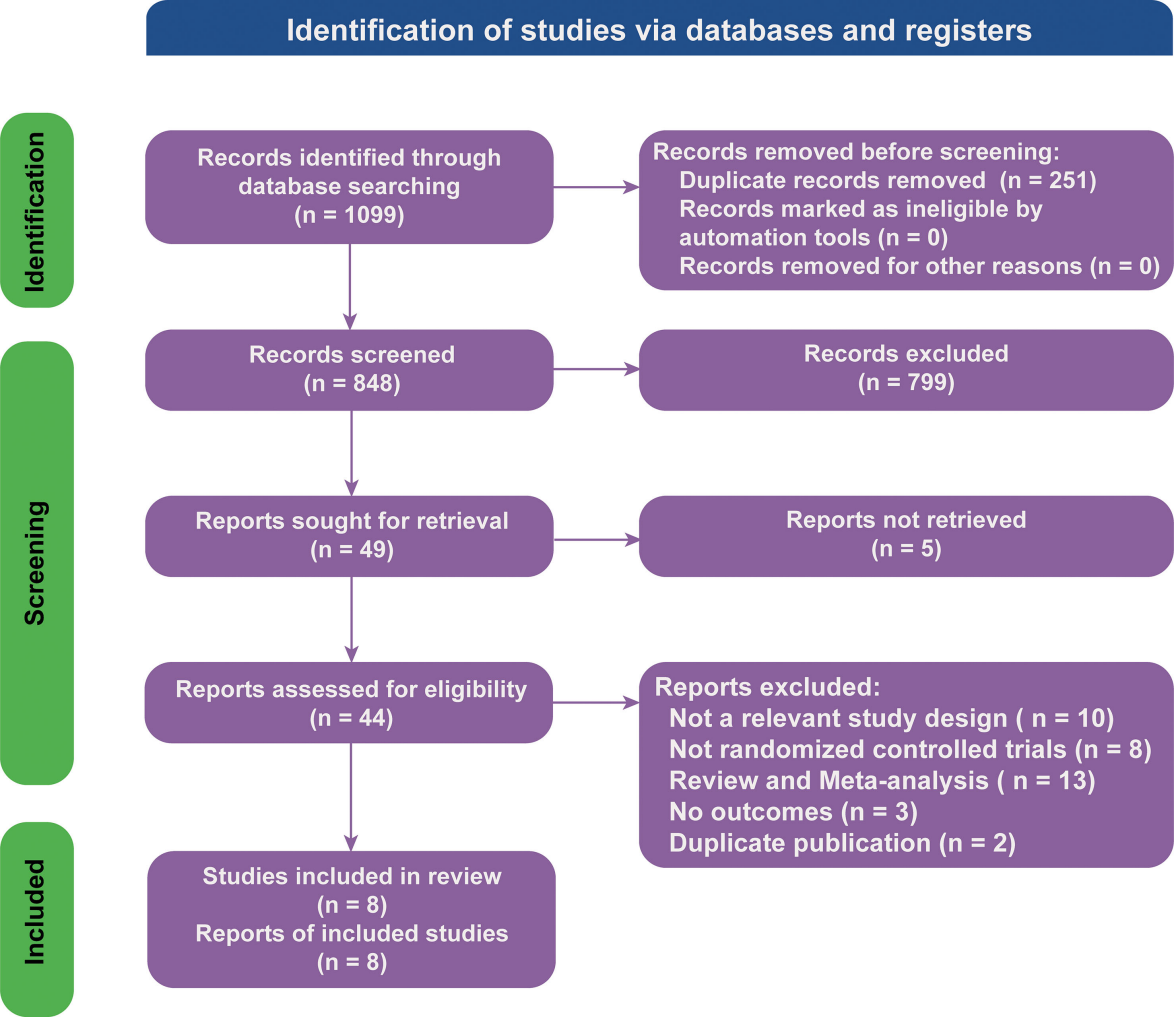
Literature search and selection. The study process followed the PRISMA guidelines.

There were 1,099 articles found in the literature search. The NMA included 8 RCTs (18, 21, 25–31) involving 4,589 participants based on an assessment of 44 full-text articles (**Figure 1**; details of included studies are shown in **Table 1**). There were four treatment regimens recorded, including the following: atezolizumab plus chemotherapy (Atezo-Chemo), durvalumab plus chemotherapy (Durva-Chemo), pembrolizumab plus chemotherapy (Pemb-Chemo), and chemotherapy (Chemo). The Risk of Bias 2 tool was used to evaluate the quality of the trial (**Supplementary Figure 1**).

**Table 1:** Baseline characteristics of studies included in the network meta-analysis.

Trial (Phase, Design)	Source (Year)	Registered ID (Randomization)	Sample Size (Median Age/y)	Participants	Treatment		Primary outcomes^a^	Secondary outcomes (ICI/chemotherapy, number [proportion])
					Experiment	Control		
IMpassion031 (III, double-blind) (25)	Lancet 2020	NCT03197935 (1:1)	165/168 (51/51)	eTNBC	Atezolizumab 840 mg Q2W plus nab-paclitaxel 125 mg/m² QW for 12 weeks, followed by atezolizumab 840 mg Q2W plus doxorubicin 60 mg/m² and cyclophosphamide 600 mg/m² Q2W for 8 weeks.	Nab-paclitaxel 125 mg/m² QW for 12 weeks, followed by doxorubicin 60 mg/m² and cyclophosphamide 600 mg/m² Q2W for 8 weeks.	PFS ITT population HR 0.74 (95% CI 0.32-1.70) OS ITT population HR 0.69 (95% CI 0.25-1.87)	pCR 95(58%)/69(41%) grade ≥ 3 trAEs 104(63%)/102(61%) Any grade irAEs 115(70%)/101(60%)
IMpassion130 (III, open-lable) (18)	NEJM 2018	NCT02425891 (1:1)	451/451 (55/56)	mTNBC	Atezolizumab 840 mg Q2W plus nab-paclitaxel 100 mg/m² QW	Nab-paclitaxel 100 mg/m² QW	PFS (1) ITT population HR 0.80 (95% CI 0.69-0.92) (2) PD-L1 positive population HR 0.62 (95% CI 0.49-0.78) OS (1) ITT population HR 0.84 (95% CI 0.69-1.02) (2) PD-L1 positive population HR 0.62 (95% CI 0.45-0.86)	pCR 32(7%)/7(1%) grade ≥ 3 trAEs 226(50%)/188(43%) Any grade irAEs 259(57%)/183(42%)
IMpassion131 (III, double-blind) (26)	ESMO 2021	NCT03125902 (2:1)	431/220 (54/53)	mTNBC	Atezolizumab 840 mg Q2W plus paclitaxel 90 mg/m² QW	Paclitaxel 90 mg/m² QW	PFS (1) ITT population HR 0.86 (95% CI 0.70-1.05) (2) PD-L1 positive population HR 0.82 (95% CI 0.60-1.12) OS (1) ITT population HR 1.12 (95% CI 0.88-1.43) (2) PD-L1 positive population HR 1.11 (95% CI 0.76-1.64)	pCR 22(5%)/11(5%) grade ≥ 3 trAEs 199(46%)/115(53%) Any grade irAEs 268(62%)/116(53%)
KEYNOTE-355 (III, double-blind) (21, 27)	Lancet 2020	NCT02819518 (2:1)	566/281 (53/53)	mTNBC	Pembrolizumab 200 mg Q3W plus nab-paclitaxel 100 mg/m² QW or paclitaxel 90 mg/m² QW or gemcitabine 1000 mg/m² plus carboplatin AUC 2	Nab-paclitaxel 100 mg/m² QW or paclitaxel 90 mg/m² QW or gemcitabine 1000 mg/m² plus carboplatin AUC 2	PFS (1) ITT population HR 0.82 (95% CI 0.69-0.97) (2) PD-L1 positive population HR 0.74 (95% CI 0.61-0.90) OS (1) ITT population HR 0.89 (95% CI 0.76-1.05) (2) PD-L1 positive population HR 0.86 (95% CI 0.72-1.04)	grade ≥ 3 trAEs 438(78%)/207(74%) Any grade irAEs 149(27%)/18(6%)
KEYNOTE-522 (III, open-lable) (28)	NEJM 2020	NCT03036488 (2:1)	784/390 (49/48)	eTNBC	Pembrolizumab 200 mg Q3W plus paclitaxel 80 mg/m² and carboplatin AUC 5 Q3W	Paclitaxel 80 mg/m² and carboplatin AUC 5 Q3W	PFS ITT population HR 0.63 (95% CI 0.43-0.93)	pCR 260(65%)/103(51%) grade ≥ 3 trAEs 633(81%)/295(76%) Any grade irAEs 304(39%)/71(18%)
SAFIR02-BREAST IMMUNO (II, open-lable) (29)	Nat Med 2021	NCT02299999 (2:1)	161/68 (56/56)	mTNBC	Durvalumab 10 mg/kg Q2W plus chemotherapy	Chemotherapy	PFS ITT population HR 0.87 (95% CI 0.54-1.42) OS ITT population HR 0.54 (95% CI 0.30-0.97)	NR^b^
GeparNuevo (II, double-blind) (30)	ESMO 2019	NCT02685059 (1:1)	88/85 (49.5/49.5)	eTNBC	Durvalumab 0.75 g 2 weeks before start of chemotherapy followed by durvalumab 1.5 g Q4W plus nabpaclitaxel 125 mg/m2 QW for 12 weeks, followed by durvalumab 1.5 g Q4W plus epirubicin/cyclophosphamide Q2W for 4 cycles.	Nabpaclitaxel 125 mg/m2 QW for 12 weeks, followed by epirubicin/cyclophosphamide Q2W for 4 cycles.	NR	pCR 44(56%)/37(46%) grade ≥ 3 trAEs 30(33%)/29(35%) Any grade irAEs 56(61%)/45(55%)
NeoTRIP Michelangelo (III, open-lable) (31)	ESMO 2022	NCT002620280 (1:1)	138/142 (49.5/50)	eTNBC	Atezolizumab 1200 mg Q3W plus nab-paclitaxel 125 mg/m2 QW and carboplatin AUC 2 on days 1 and 8 Q3W	Nab-paclitaxel 125 mg/m2 QW plus carboplatin AUC 2 on days 1 and 8 Q3W	NR	pCR 67(49%)/63(44%) grade ≥ 3 trAEs 107(78%)/98(70%)

AUC, area under the curve; CI, confidence interval; HR, hazard ratio; ICI, immune checkpoint inhibitor; ITT, intention-to-treat; QW, every week; Q2W, every two weeks; Q3W, every three weeks; Q4W, every four weeks.

a The results was ICIs compared with chemotherapy.

b Due to those results in SAFIR02-BREAST IMMUNO trial included other type of breast cancer patients, we did not included those results.

### Comparisons of progression-free survival

**Figure 2:**
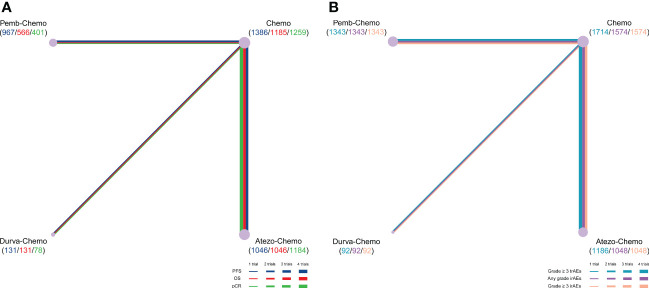
Comparative network plots for efficacy and toxicity of immunotherapy in patients with TNBC. Lines represent trials comparing 2 drugs or drugs for different outcomes. The nodes indicate the drug treatments assessed in existing trials. The size of the node and the width of the line are proportional to the number of randomized controlled trials and comparisons, respectively. Comparisons were generated by using the Bayesian framework on **(A)** PFS, OS, pCR, **(B)** any-grade trAEs, any-grade irAEs and grade ≥ 3 irAEs. Atezo, atezolizumab; Chemo, chemotherapy; Durva, durvalumab; irAEs, immune-related adverse events; OS, overall survival; pCR, pathologic complete response; Pemb, pembrolizumab; PFS, progression-free survival; TNBC, triple-negative breast cancer; trAEs, treatment-related adverse events.

**Figure 3:**
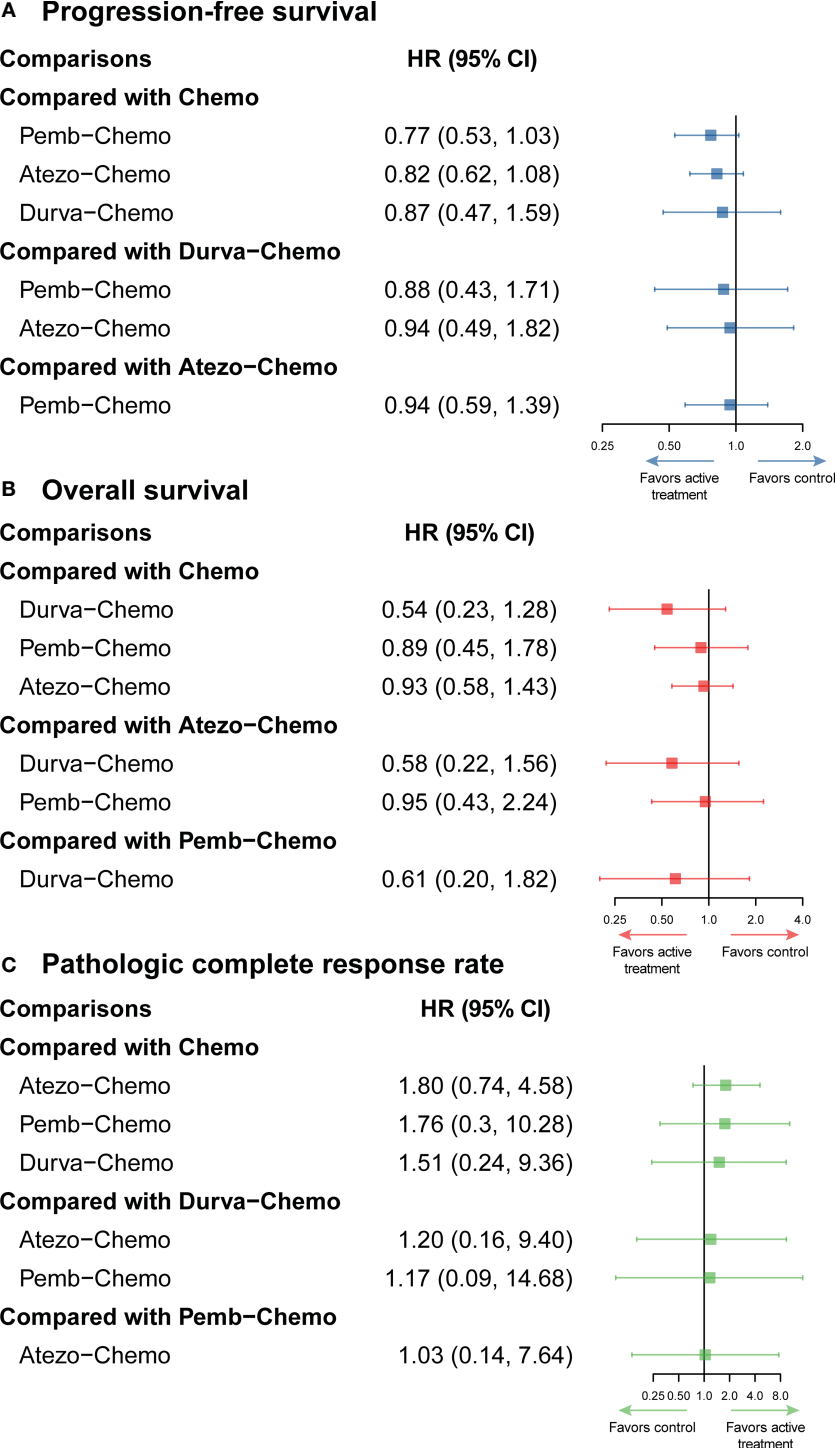
Efficacy profiles of the Bayesian network meta-analysis in patients with TNBC. **(A)** forest plot of progression-free survival; **(B)** forest plot of overall survival; **(C)** forest plot of pathologic complete response. Atezo, atezolizumab; Chemo, chemotherapy; CI confidence interval; Durva, durvalumab; HR, hazard ratio; Pemb, pembrolizumab; TNBC, triple-negative breast cancer.

**Figure 4:**
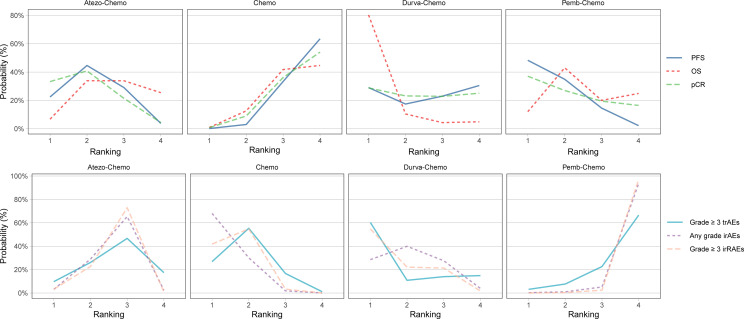
Bayesian ranking profiles for ICIs on efficacy and safety for patients with TNBC. Ranking plots indicate the probability of each comparable immunotherapy combination being ranked from first to last on PFS, OS, pCR, any-grade trAEs, any-grade irAEs and grade ≥ 3 irAEs. The graphs display the distribution of probabilities of treatment ranking from best through worst for each outcome. Ranking indicates the probability that drug class is first “best,” second “best,” etc. Atezo, atezolizumab; Chemo, chemotherapy; Durva, durvalumab; ICI, immune checkpoint inhibitor; irAEs, immune-related adverse events; OS, overall survival; pCR, pathologic complete response; Pemb, pembrolizumab; PFS, progression-free survival; TNBC, triple-negative breast cancer; trAEs, treatment-related adverse events.

NMA included 4 treatments for PFS in patients with TNBC (**Figure 2**). No significant differences were found among the treatment regimens in the ITT population for improvement of PFS (**Figure 3**). Based on Bayesian ranking profiles, Pemb-Chemo was ranked first for PFS, followed by Atezo-Chemo and Durva-Chemo (**Figure 4**).

A specific subgroup analysis for the PFS endpoint was conducted in the PD-L1 positive population (**Supplementary Figure 2**). Similar results revealed that no significant improvement was found among the treatment agents in the PD-L1-positive population (**Supplementary Figure 3**). Based on Bayesian ranking profiles, Atezo-Chemo was ranked first for PFS, followed by Pemb-Chemo (**Supplementary Figure 4**). Further subgroup analysis was performed in patients with metastatic TNBC, and the results showed no significant improvement among the comparisons (**Supplementary Figure 5**).

### Comparisons of overall survival

NMA included 4 treatments for OS in patients with TNBC (**Figure 2**). All of the evaluated comparisons for OS were statistically non-significant in the ITT population (**Figure 3**). Based on Bayesian ranking profiles, Durva-Chemo was ranked first for OS, followed by Pemb-Chemo and Atezo-Chemo (**Figure 4**).

A specific subgroup analysis was carried out in the PD-L1 positive population, the HRs of OS were not statistically significant among the comparison (**Supplementary Figure 6**). Based on Bayesian ranking profiles, Atezo-Chemo was ranked first for OS, followed by Pemb-Chemo (**Supplementary Figure 4**). Further subgroup analysis was performed in patients with metastatic TNBC, and the results showed no significant improvement among the comparisons (**Supplementary Figure 7**).

### Comparisons of pathologic complete response rate

The pCR rate was analyzed in 6 studies with 4 treatments in patients with TNBC (**Figure 2**). However, the results presented no significance in the comparison of the ITT population (**Figure 3**). Based on Bayesian ranking profiles, Atezo-Chemo was ranked first for pCR, followed by Pemb-Chemo and Durva-Chemo (**Figure 4**).

The subgroup analysis was performed in the PD-L1 positive population and the favorable tendency of ICI for the treatment of pCR; however, the results showed no significantly different (**Supplementary Figure 8**). Based on Bayesian ranking profiles, Atezo-Chemo was ranked first for pCR, followed by Pemb-Chemo and Durva-Chemo (**Supplementary Figure 4**).

### Safety analysis

Overall, 7 studies including 4335 patients were included in the safety analysis (**Figure 2**).

**Figure 5:**
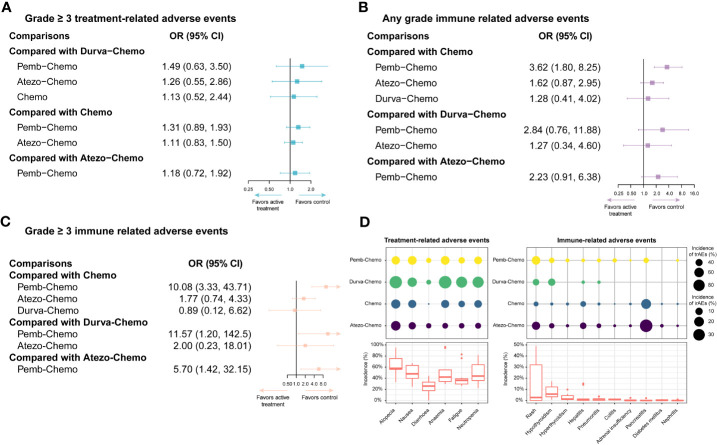
Safety profiles of the Bayesian network meta-analysis in patients with TNBC. **(A)** forest plot of any-grade trAEs; **(B)** forest plot of any-grade irAEs; **(C)** forest plot of grade ≥ 3 irAEs; **(D)** incidence of treatment-related and immune-related AEs. Atezo, atezolizumab; Chemo, chemotherapy; CI confidence interval; Durva, durvalumab; irAEs, immune-related adverse events; OS, overall survival; HR, hazard ratio; pCR, pathologic complete response; Pemb, pembrolizumab; PFS, progression-free survival; TNBC, triple-negative breast cancer; trAEs, treatment-related adverse events.

In accordance with the safety analysis, an analysis of grade ≥ 3 trAEs has been conducted. No significant difference was found among the treatment agents in grade ≥ 3 trAEs (**Figure 5**). Based on Bayesian ranking profiles, Pemb-Chemo was ranked worst for grade ≥ 3 trAEs, followed by Atezo-Chemo (**Figure 4**).

In terms of any grade irAEs, compared with Chemo, Pemb-Chemo was related higher incidence of irAEs and the result was statistically significant (**Figure 5**). Based on Bayesian ranking profiles, Pemb-Chemo was ranked worst for any grade irAEs, followed by Atezo-Chemo and Durva-Chemo (**Figure 4**).

In terms of grade ≥ 3 irAEs, compared with Chemo, Durva-Chemo, and Atezo-Chemo, Pemb-Chemo was significantly related to a higher incidence of grade ≥ 3 irAEs (**Figure 5**). Based on Bayesian ranking profiles, Pemb-Chemo was ranked worst for grade ≥ 3 irAEs, followed by Atezo-Chemo and Durva-Chemo (**Figure 4**).

We conducted a comprehensive analysis of the included studies in order to estimate the frequency of certain trAEs and irAEs. The most frequent trAEs were alopecia (60.20% with Atezo-Chemo, 48.92% with Pemb-Chemo and 92.93% with Durva-Chemo) followed by nausea and anemia (**Figure 5**). There was a relatively uniform frequency of the most common trAEs of any grade between the treatment arms (**Figure 5**). In addition, the most common irAEs associated with ICI were hypothyroidism (13.24% with Atezo-Chemo, 14.59% with Pemb-Chemo, and 7.61% with Durva-Chemo) followed by hyperthyroidism (**Figure 5**).

## Discussion

To the best of our knowledge, this is the first NMA to evaluate the efficacy and safety profiles of currently available ICI treatments for patients with TNBC. As part of our study, we aimed to discuss the potential clinical implications of ICIs in this setting as well as some controversial aspects, such as PD-L1 positive patients. A controversial issue is whether immunotherapy can enhance the therapeutic effect of original standard chemotherapy (8, 9). The present study evaluated ICIs in combination with chemotherapy as the first-line treatment for TNBC in all included studies. Concomitant chemotherapy has been shown to enhance the anticancer effect of ICI by increasing the release of cancer antigens (32). In spite of this, the question of which immunomodulatory therapy will be more effective when combined with chemotherapy remains an open question. Several research studies are underway in an effort to find treatments that mobilize and activate antitumor T cells and/or move immune suppression in the direction of immune activation.

Considering this background, to determine the efficacy and safety of ICIs in patients with TNBC, a systematic review and NMA of RCTs were performed. This NMA yielded two key findings regarding the efficacy and safety of ICIs for the treatment of TNBC. First, ICIs plus chemotherapy showed a trend toward better outcomes in terms of PFS, OS, and pCR, especially for the PD-L1 positive population. However, the results of this study were shown no significant difference among the comparison. It is clear that ICI plus chemotherapy had a trend of better survival advantage than chemotherapy alone as survival increased with the extension of OS and PFS. There was a higher incidence of grade ≥ 3 trAEs, any grade irAEs, and grade ≥ 3 irAEs in the ICI plus chemotherapy group compared to the control group. Second, Bayesian ranking is a useful tool to rank the probability of each treatment. Based on the Bayesian ranking profile, Atezo-Chemo was related to higher rank probability in terms of PFS, OS, and pCR in the PD-L1 positive population. Pemb-Chemo was associated with a relatively higher probability in terms of PFS, OS, and pCR in the ITT population. As for the safety profile, Pemb-Chemo was related relatively to higher risk in terms of grade ≥ 3 trAEs any grade irAEs, and grade ≥ 3 irAEs, followed by Atezo-Chemo. Therefore, we suggested that Pemb-Chemo and Atezo-Chemo are better with longer survival for ITT TNBC population or PD-L1 positive TNBC population; however, those treatments had less favorable safety profiles. Hence, ICIs plus chemotherapy should be used cautiously. Based on the results, it appears that the combination of ICIs and chemotherapy trend to the better OS, PFS, and pCR rates. Immunotherapeutic approaches to TNBC are justified by a number of factors. There are two possible explanations for the benefit of ICIs in combination with chemotherapy. First, it is important to note that ICIs kill tumor cells by activating tumor immunity, which is different from chemotherapy and plays a synergetic role in the treatment of TNBC, especially in PD-L1 positive patients (28, 33). Second, in early breast cancer, the antitumor effect may be more significant than in metastatic disease due to the robustness of the tumor’s immune microenvironment (34).

Several traditional pairwise meta-analyses that evaluated the efficacy and safety profiles of ICI for patients with TNBC have been published. It has been reported in two previous publications that ICIs added to chemotherapy are associated with improved PFS and OS in TNBC patients with PD-L1 positivity, and those findings were similar to our findings. ICIs plus chemotherapy appears to be better than chemotherapy alone for TNBC treatment, with better OS and PFS, especially for PD-L1 positive population, and its high rates of serious AEs need to be taken seriously (35, 36). In another study, PD-1 and PD-L1 inhibitors were combined with chemotherapy as a treatment for TNBC (37). The results of this study demonstrated a significant pCR benefit and confirmed that PD-1/PD-L1 ICIs plus chemotherapy may improve the PFS of PD-1/PD-L1 patients in both neoadjuvant and adjuvant settings, with tolerable safety events. In the last study, platinum-based chemotherapy and immunotherapy were evaluated in early TNBC, and the results showed that ICIs plus chemotherapy increased the pCR rate and reduced adverse effects when compared to platinum-based chemotherapy (38).

There are several differences between the previously published and the current publication mainly in the following aspects. First, our study is an NMA, standard pairwise meta-analysis is limited to comparing two drug categories that have been evaluated in head-to-head trials. Several treatment options exist for a complex condition, many of which have not been directly compared in clinical trials. The theoretical advantage of NMA is based on the integration of direct evidence from studies directly comparing a particular treatment comparison with indirect evidence from pathways with at least one intermediate comparator within a single framework that ranks treatments by efficacy and safety (39). Second, compared with chemotherapy alone, single ICIs plus chemotherapy showed a trend of benefit without significant differences. Third, subgroup analyses of PD-L1-positive TNBC patients treated with ICIs plus chemotherapy also showed a trend of benefit with statistically non-significant. Fourth, our study included and analyzed both early and metastatic TNBC. Lastly, the discrepancies between trAEs and irAEs in these treatments were examined in detail in order to identify those AEs that require additional attention when they are combined with ICIs.

### Implications

The purpose of this study is to summarize the findings of RCTs in order to provide clinicians with a reference source to evaluate the strengths and weaknesses of several promising options that are available for practice. When taking both efficacy and safety into consideration, Pemb-Chemo and Atezo-Chemo seem to be superior first-line treatments for patients with TNBC with ITT and PD-L1 positive, respectively. Nevertheless, there is a need for further trials in which head-to-head comparisons are conducted, such as Pemb-Chemo versus Atezo-Chemo versus Durva-Chemo or ICIs monotherapy. In addition, these findings may help answer the question of whether ICIs should be included in the standard care of patients with TNBC and which treatment is most appropriate for those with no actionable mutations.

### Limitations

This study has some limitations that need to be addressed. First, we analyzed published results rather than individual patient data. Second, In the included trials, the method of assessing PD-L1 was different. For example, whereas PD-L1 was evaluated in the KEYNOTE 355 trial by IHC 22C3 pharmDx assay and characterized by CPS, PD-L1 positivity in IMpassion trials was determined by immune cell staining of 1% in accordance with VENTANA PD-L1 SP142 immunohistochemical testing. As a result, it is necessary to harmonize PD-L1 testing across clinical trials in order to address this issue in clinical studies of immunotherapy for both early and metastatic breast cancer. This suggests that it is prudent to interpret the aggregate findings of PD-L1 positive populations cautiously. Third, PFS and OS were not primary outcomes in GeparNuevo and NeoTRIP Michelangelo trials and subgroup analysis data for PFS and OS has not been reported for half of the included trials. Therefore, in order to strengthen the results of this study, future studies should include RCTs with a greater number of participants. Fourth, patients with TNBC who lack genetic alterations can normally be treated with taxane or platinum-based chemotherapy as a first-line standard systemic treatment. The chemotherapeutic strategies in this study were varied and included both taxane and platinum chemotherapy. The combination of different chemotherapy regimens with immunotherapy may therefore have different synergistic effects. Finally, the 95% intervals generated by the Bayesian approach are slightly wider than those under the traditional pairwise meta-analysis a function of the prior used. While the size of the discrepancies was heavily dependent on the number of studies available and the amount of heterogeneity in the data, another key driver for the difference was the “prior information” used in the Bayesian analyses for random-effects variance. In this study, due to the limitation of the number of included studies, no statistically significant differences in most of outcomes. So generally, little is lost in basing conclusions about the treatment comparisons on the potentially more conservative 95% intervals generated by the Bayesian approach.

## Conclusion

In summary, our study demonstrated the efficacy and safety of different ICIs in the treatment of TNBC. When taking both efficacy and safety into consideration, Pemb-Chemo and Atezo-Chemo seem to be superior first-line treatments for patients with TNBC with ITT and PD-L1-positive populations, respectively. Nevertheless, both ICIs plus chemotherapy schedules were associated with a higher risk of trAEs or irAEs. It may be useful for making clinical decisions based on this information.

## Data availability statement

The raw data supporting the conclusions of this article will be made available by the authors, without undue reservation.

## Author contributions

Study concept and design: XL and YL. Data acquisition and management: XL and XC. Statistical analysis: XL and XC. Interpretation of data: HL. Drafting of the manuscript: XL. Critical revision of the manuscript for important intellectual content: XC and YL. Study supervision: YL and HL.

## Supplementary material

Supplementary Figure 1 Assessment of Risk of Bias using Cochrane Risk of Bias Tool 2.0.

Supplementary Figure 2 Comparative network plots for efficacy of ICIs in patients with PD-L1-positive TNBC.

Supplementary Figure 3 Forest plot of progression-free survival of ICIs in patients with PD-L1-positive TNBC.

Supplementary Figure 4 Bayesian ranking profiles for ICIs on efficacy and safety for patients with PD-L1-positive TNBC.

Supplementary Figure 5 Forest plot of progression-free survival of ICIs in patients with metastatic TNBC.

Supplementary Figure 6 Forest plot of overall survival of ICIs in patients with PD-L1-positive TNBC.

Supplementary Figure 7 Forest plot of overall survival of ICIs in patients with metastatic TNBC.

Supplementary Figure 8 Forest plot of pathologic complete response rate of ICIs in patients with PD-L1-positive TNBC.
