# Supplementary material for: Immune checkpoint inhibitors in first-line therapies of metastatic or early triple-negative breast cancer: a systematic review and network meta-analysis
Source: Front Endocrinol (Lausanne). 2023 May 9;14:1137464. doi: 10.3389/fendo.2023.1137464 (PMC10204114; doi:10.3389/fendo.2023.1137464)
Supplement: Supplementary Figure 1 — Assessment of Risk of Bias using Cochrane Risk of Bias Tool 2.0. [file DataSheet_1.docx]

**Supplementary Content**

**Supplementary Figure 1.** Assessment of Risk of Bias using Cochrane Risk of Bias Tool 2.0.

**Supplementary Figure 2.** Comparative network plots for efficacy of ICIs in patients with PD-L1-positive advanced TNBC.

**Supplementary Figure 3**. Forest plot of progression-free survival of ICIs in patients with PD-L1-positive advanced TNBC.

**Supplementary Figure 4**. Bayesian ranking profiles for ICIs on efficacy and safety for patients with PD-L1-positive advanced TNBC.

**Supplementary Figure 5.** Forest plot of progression-free survival of ICIs in patients with metastatic TNBC.

**Supplementary Figure 6.** Forest plot of overall survival of ICIs in patients with PD-L1-positive advanced TNBC.

**Supplementary Figure 7.** Forest plot of overall survival of ICIs in patients with metastatic TNBC.

**Supplementary Figure 8.** Forest plot of pathologic complete response rate of ICIs in patients with PD-L1-positive advanced TNBC.


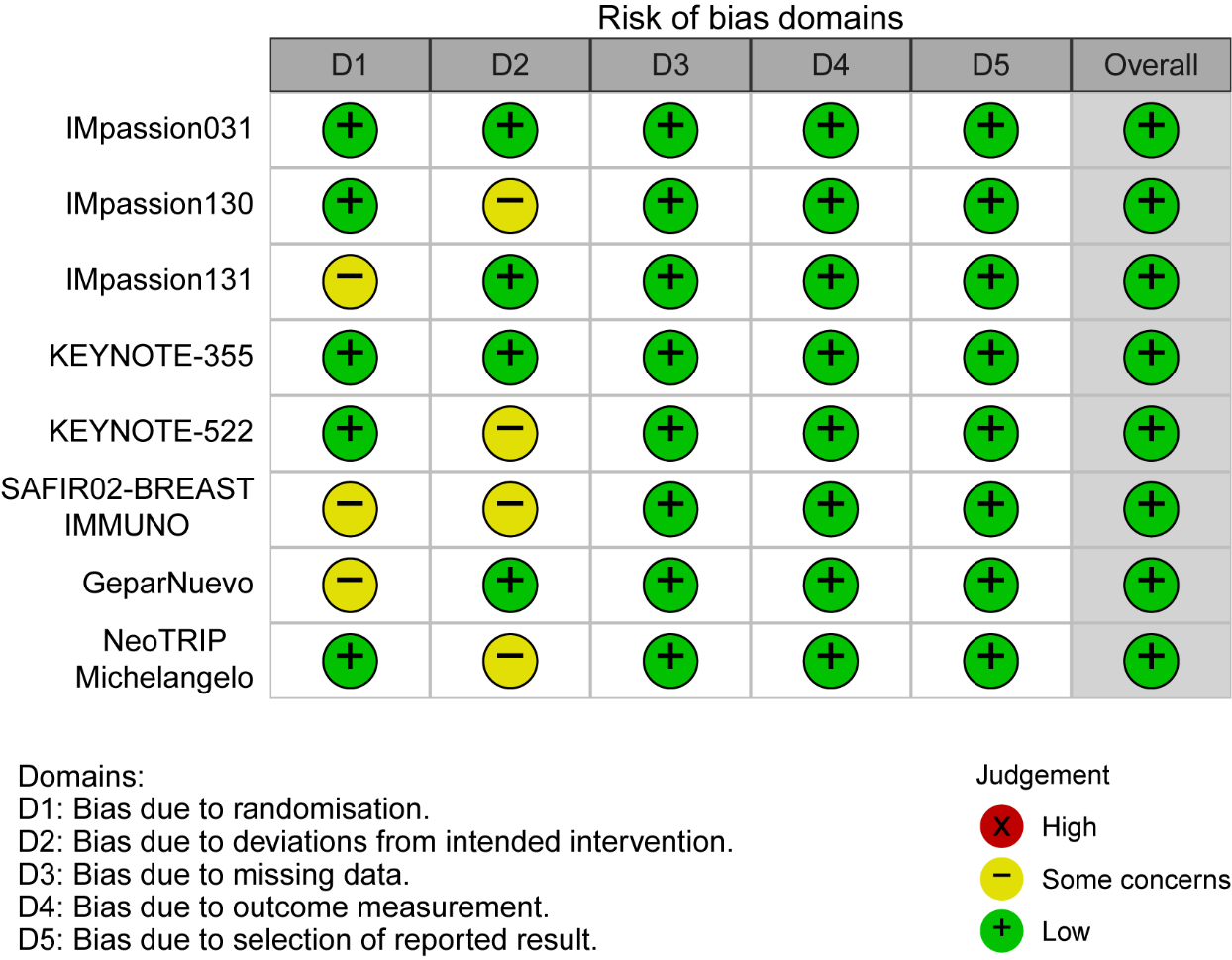


**Supplementary Figure 1.** Assessment of Risk of Bias using Cochrane Risk of Bias Tool 2.0


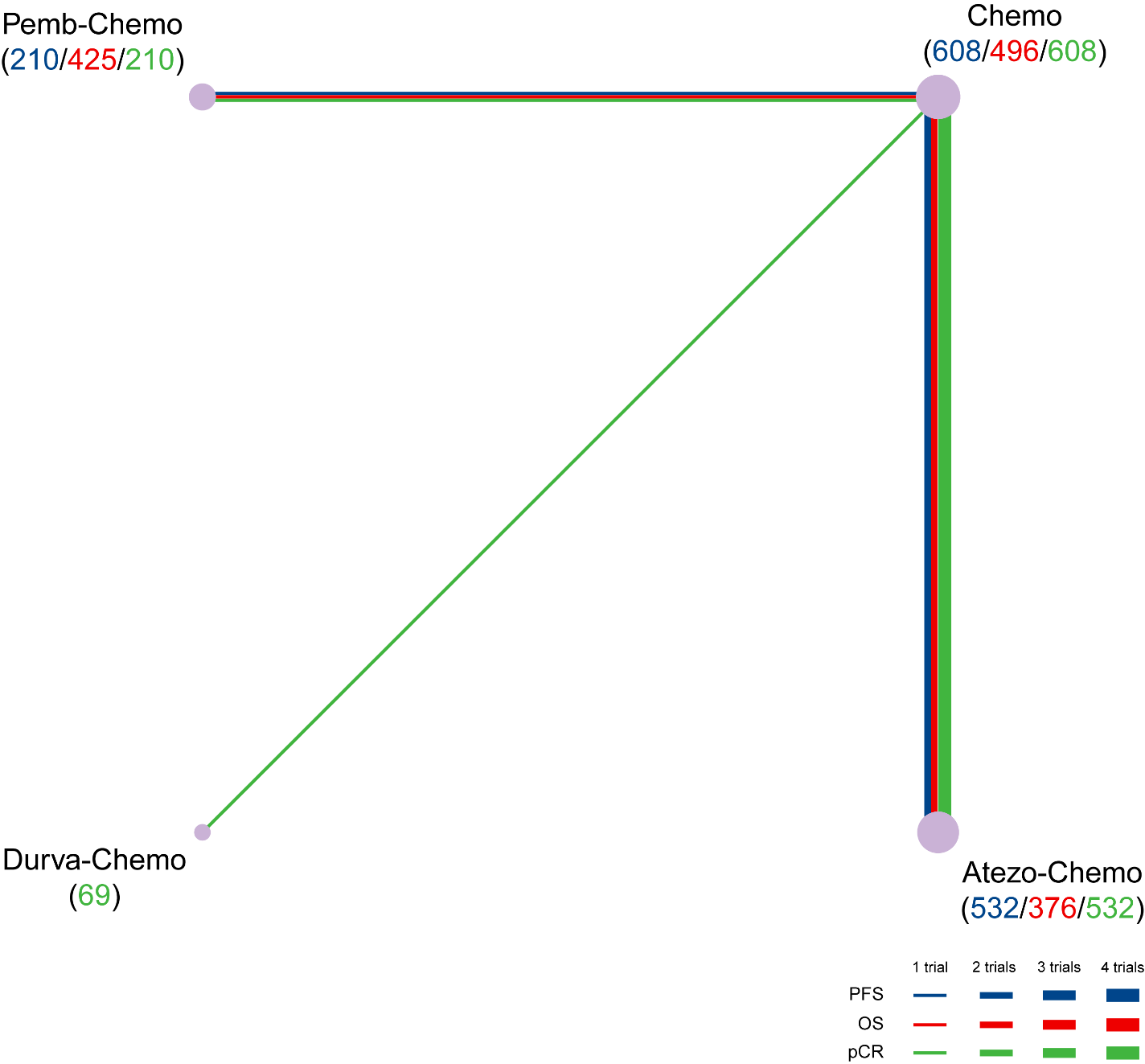


**Supplementary Figure 2.** Comparative network plots for efficacy of ICIs in patients with PD-L1-positive advanced TNBC. Each circle represents an intervention as a node in the network. The size of the circle and the width of the line are proportional to the number of randomized controlled trials and comparisons, respectively. Atezo, atezolizumab; Chemo, chemotherapy; Durva, durvalumab; ICI, immune checkpoint inhibitor; OS, overall survival; pCR, pathologic complete response; Pem, pembrolizumab; PD-L1, programmed death-ligand 1; PFS, progression-free survival.


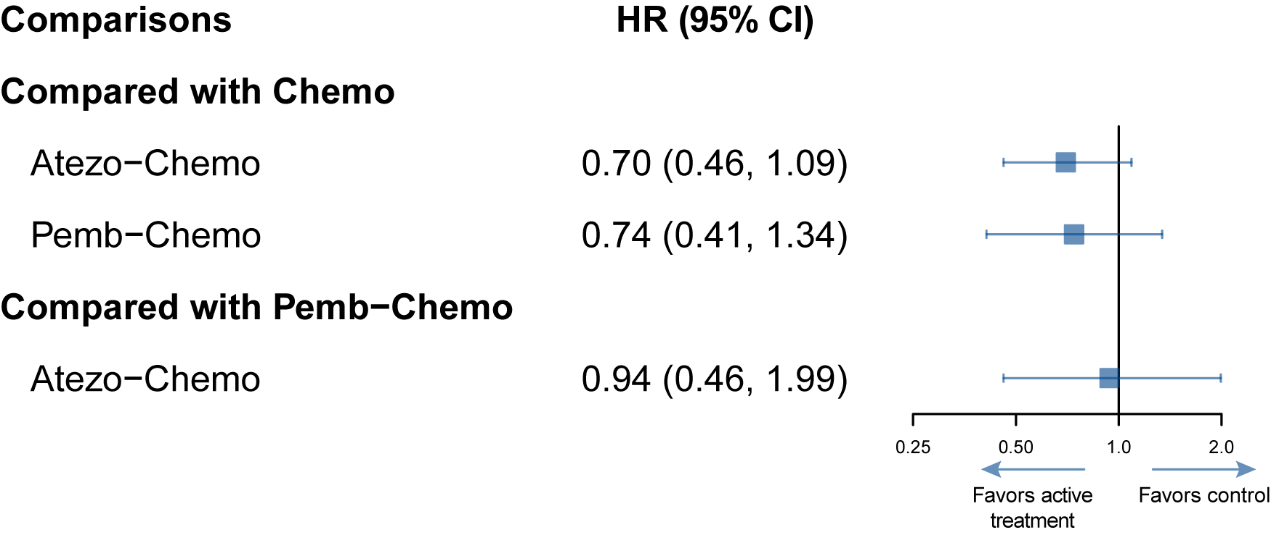


**Supplementary Figure 3**. Forest plot of progression-free survival of ICIs in patients with PD-L1-positive advanced TNBC. Atezo, atezolizumab; Chemo, chemotherapy; CI confidence interval; Durva, durvalumab; HR, hazard ratio; PD-L1, programmed death-ligand 1; Pem, pembrolizumab.


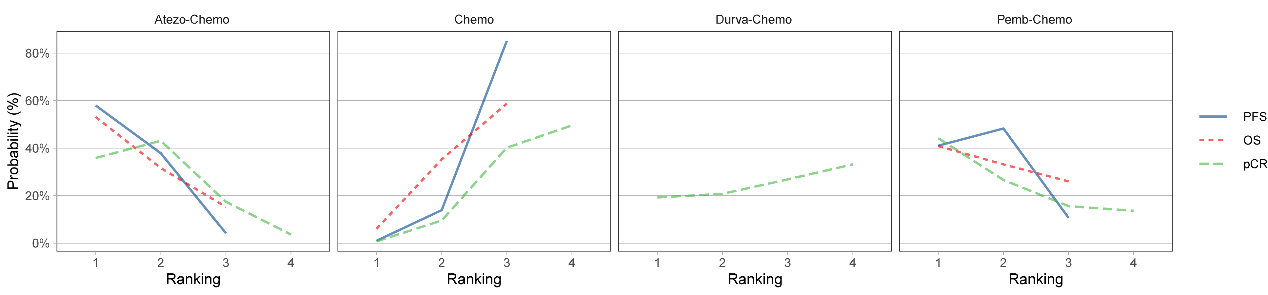


**Supplementary Figure 4**. Bayesian ranking profiles for ICIs on efficacy and safety for patients with PD-L1-positive advanced TNBC. Atezo, atezolizumab; Chemo, chemotherapy; Durva, durvalumab; ICI, immune checkpoint inhibitor; OS, overall survival; pCR, pathologic complete response; PD-L1, programmed death-ligand 1; Pem, pembrolizumab; PFS, progression-free survival; TNBC, triple-negative breast cancer.


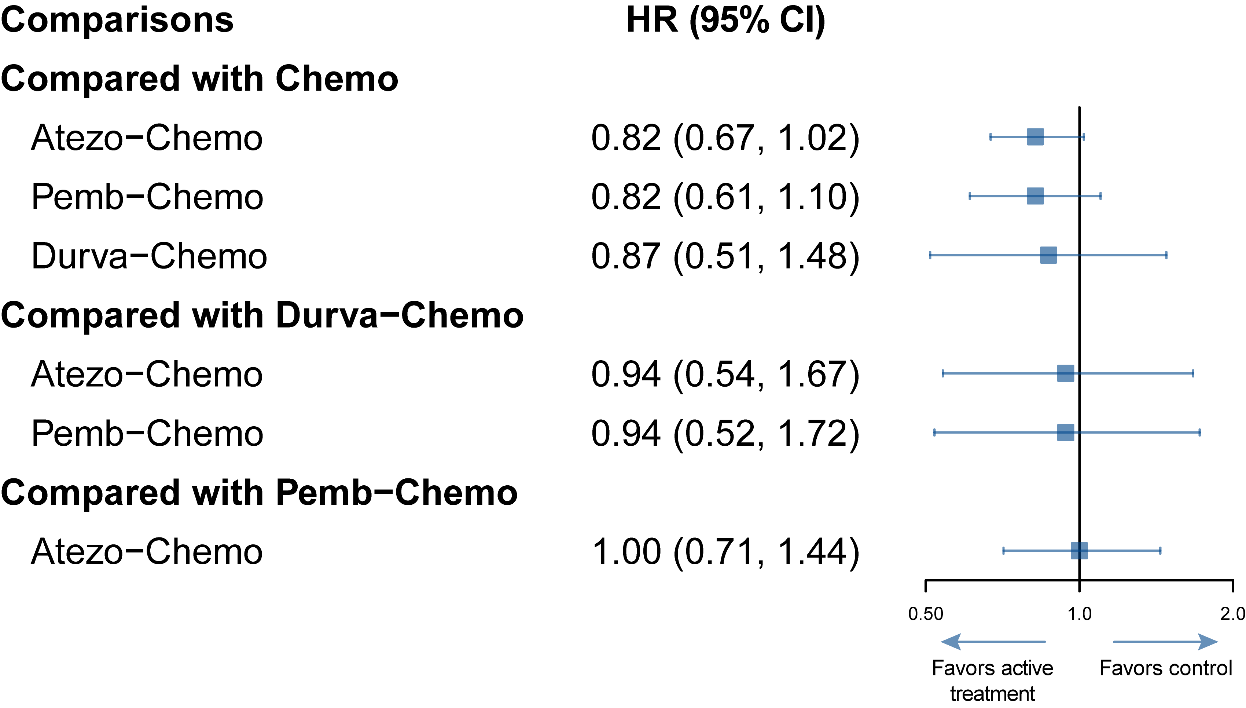


**Supplementary Figure 5**. Forest plot of progression-free survival of ICIs in patients with metastatic TNBC. Atezo, atezolizumab; Chemo, chemotherapy; Durva, durvalumab; CI confidence interval; HR, hazard ratio; Pem, pembrolizumab.


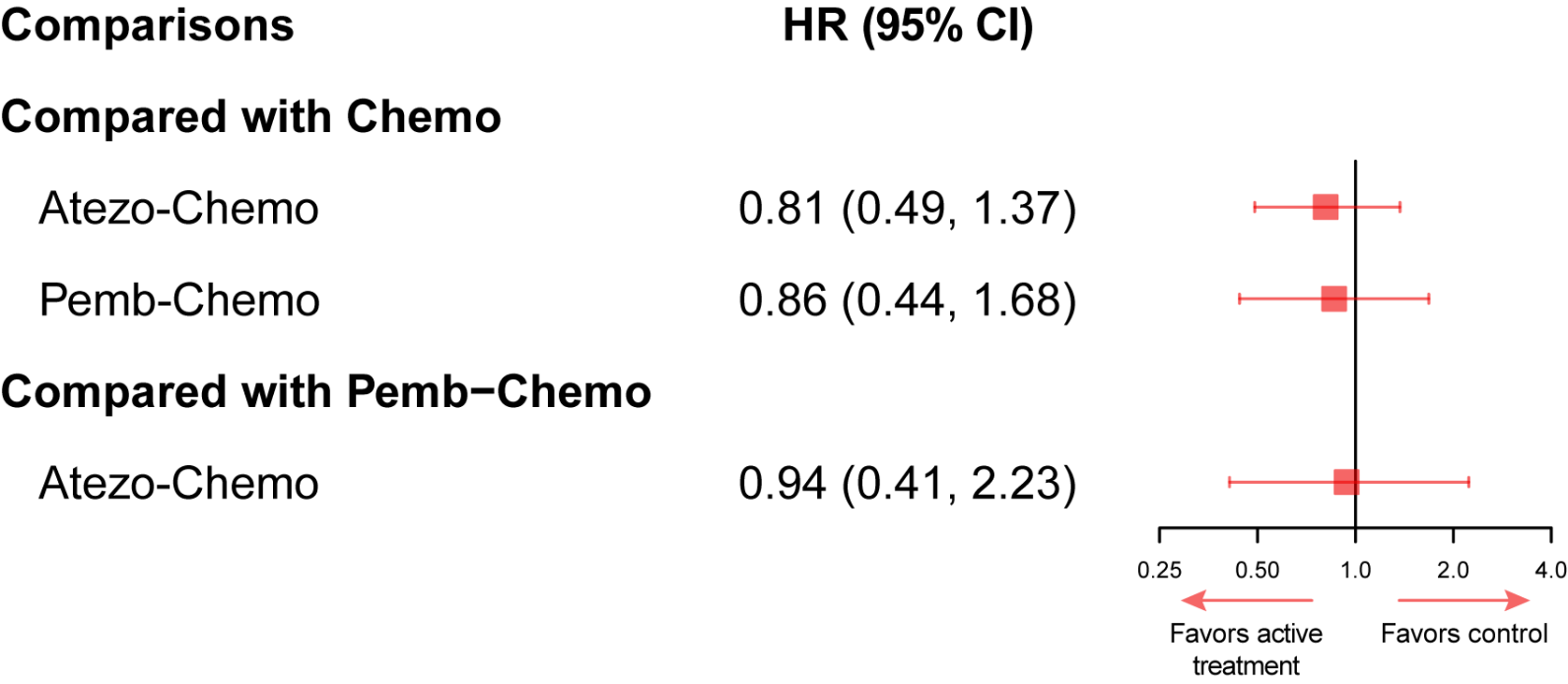


**Supplementary Figure 6**. Forest plot of overall survival of ICIs in patients with PD-L1-positive advanced TNBC. Atezo, atezolizumab; Chemo, chemotherapy; CI confidence interval; HR, hazard ratio; PD-L1, programmed death-ligand 1; Pem, pembrolizumab.


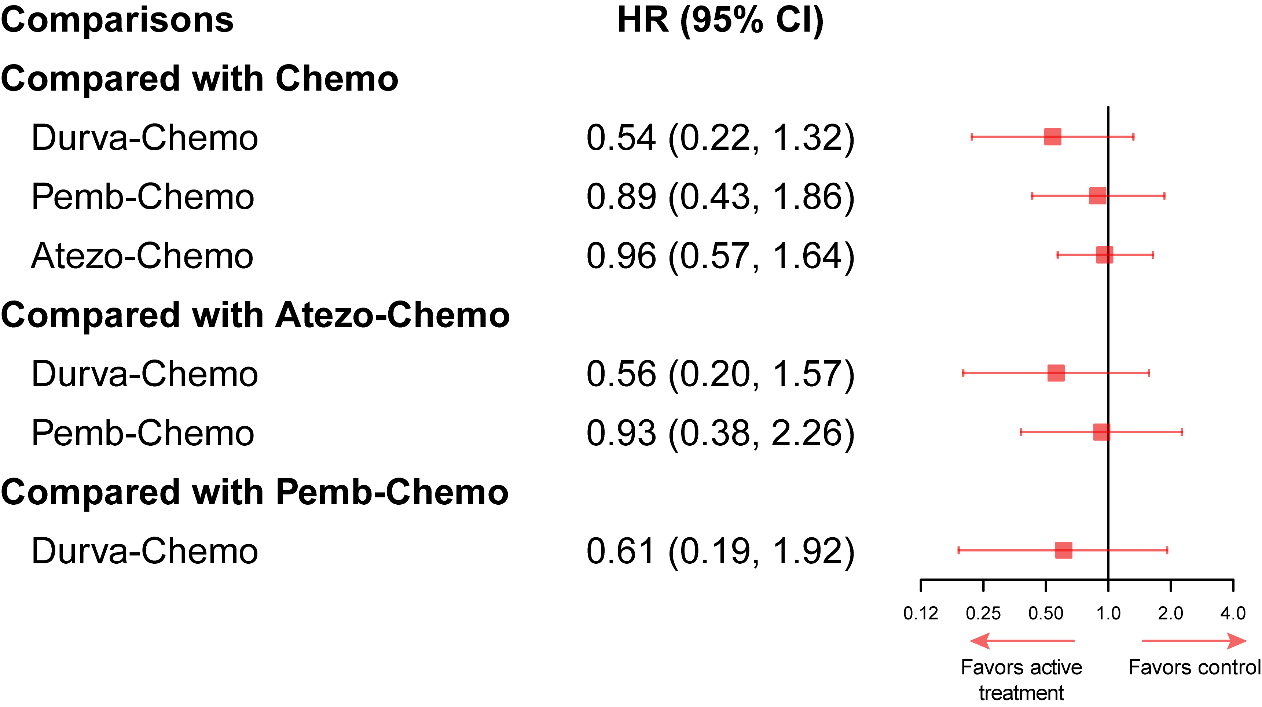


**Supplementary Figure 7**. Forest plot of overall survival of ICIs in patients with metastatic TNBC. Atezo, atezolizumab; Chemo, chemotherapy; Durva, durvalumab; CI confidence interval; HR, hazard ratio; Pem, pembrolizumab.


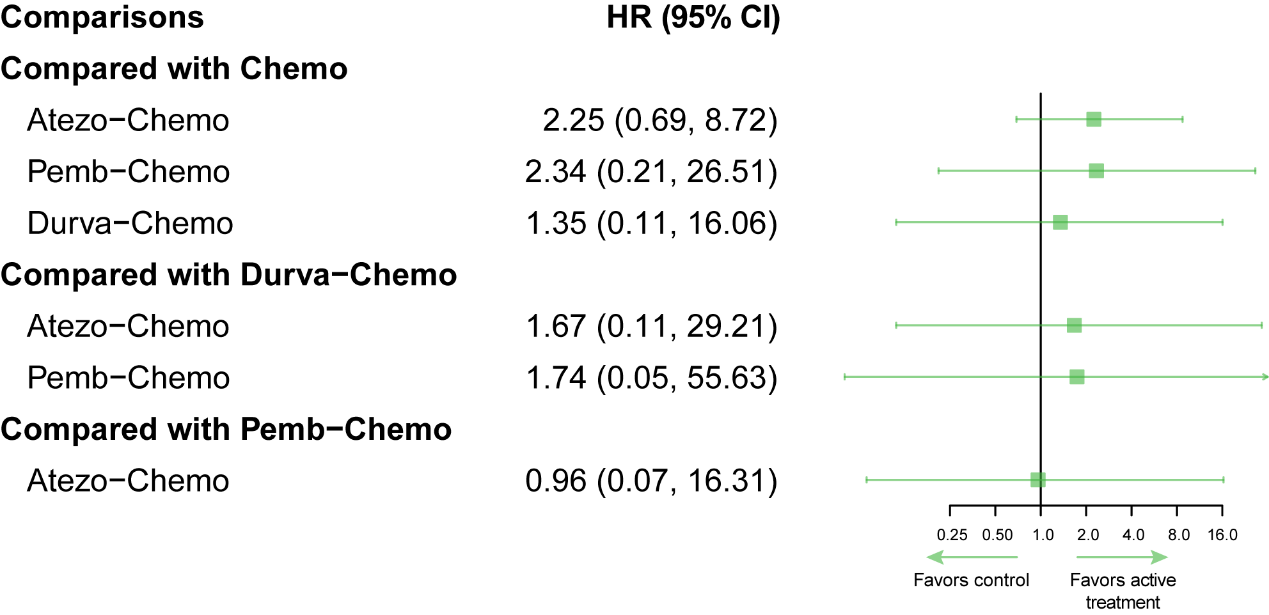


**Supplementary Figure 8**. forest plot of pathologic complete response rate of ICIs in patients with PD-L1-positive advanced TNBC. Atezo, atezolizumab; Chemo, chemotherapy; CI confidence interval; Durva, durvalumab; HR, hazard ratio; PD-L1, programmed death-ligand 1; Pem, pembrolizumab.
